# Electrochemical Immunosensor Based on CS@AuNPs/ZIF-8/rGO Composite for Detecting CA15-3 in Human Serum

**DOI:** 10.3390/s25247462

**Published:** 2025-12-08

**Authors:** Yuanyue Lu, Yong Mei, Yingying Gu, Ye Tao, Yuhan Yang, Jiao Yu, Yang Zhang, Lin Liu, Xin Li

**Affiliations:** Hubei Province Key Laboratory of Occupational Hazard Identification and Control, Department of Medical Sciences, Wuhan University of Science and Technology, Wuhan 430000, China; lyy0128@wust.edu.cn (Y.L.);

**Keywords:** metal–organic frameworks, gold nanoparticles, immunosensor, breast cancer tumor marker, CA15-3

## Abstract

An electrochemical immunosensor was fabricated to identify CA15-3, a biomarker for breast cancer (BC). A composite sensor substrate made of “zeolitic imidazolate framework-8” (ZIF-8) and “reduced graphene oxide” (rGO) was chosen and its conductivity was further improved by the addition of chitosan (CS)-doped gold nanoparticles (AuNPs). The CS@AuNPs are able to conjugate with antibodies via the strong Au-S interaction, which offers multiple active sites for antibody immobilization and enhances the sensor performance. This immunosensor is capable of ultrasensitive detection of CA15-3 by specific antigen–antibody –interactions. In healthy people, normal serum CA15-3 is up to 25 U/mL. Under optimized experimental conditions, the alteration in the signal intensity measured by the sensor was related to the CA15-3 activity. The quantitative relationship was linear over 0.001–400 U/mL with a limit of detection (LOD) of 0.0031 U/mL at a “signal-to-noise ratio” (S/N) of 3 and a “correlation coefficient” (r^2^) of 0.9983. The developed immunosensor showed great accuracy, stability, and selectivity, and was able to detect CA15-3 in human serum samples. These results validate its potential as a reliable analytical platform for BC diagnosis and early clinical screening.

## 1. Introduction

Breast cancer (BC) is a leading cause of cancer-related deaths with important global health impact for women and continues to demonstrate a growing disease burden [1]. Epidemiological research shows that about 30% of all new diagnosed cancers in women are BC, which is the highest-incidence cancer among women, and the mortality rate continues to rise [2,3].

Carbohydrate antigen 15-3 (CA15-3) is a widely used serum tumor marker in the clinical management of breast cancer. Its clinical usefulness is most apparent in the longitudinal assessment of patients with advanced disease, where serial changes in CA15-3 can mirror tumor burden [4,5]. Numerous studies have shown that circulating CA15-3 levels correlate with overall tumor load, with reported specificities of up to 97% in metastatic breast cancer [6,7]. In healthy individuals, serum CA15-3 values are typically below 25 U/mL, and activities above this cut-off raise suspicion for an underlying malignancy, including breast cancer [8]. Despite this limitation, CA15-3 is still widely employed to track treatment response and to detect disease recurrence or distant dissemination [9]. Consequently, sensitive and reliable dynamic measurement of low-abundance CA15-3 is important for supporting auxiliary diagnosis, evaluating disease course, and ultimately helping to reduce breast cancer-related morbidity and mortality.

Current diagnostic methods for breast cancer biomarkers include immunohistochemistry (IHC), enzyme-linked immunosorbent assay (ELISA), and fluorescence in situ hybridization (FISH), among others [10,11]. However, these techniques generally suffer from drawbacks such as time-consuming procedures, limited sensitivity, and the risk of false-positive or false-negative results. In addition, some of these diagnostic approaches may be invasive and potentially detrimental to patients’ health.

In contrast, electrochemical biosensors have unique advantages such as simplicity, rapidity, high sensitivity, ease of operation, scalability, and low cost. These characteristics make them a promising technology for biomarker detection of cancer. Quantitative measurement of biomarkers makes it possible to detect and classify tumors early [12,13,14]. In recent years, driven by advances in nanotechnology and sensing technology, research on CA15-3 immunosensors has progressed markedly. Various configurations have been reported, including label-free and label-based formats, enzyme-amplified systems, and electrochemical platforms enhanced by functional nanomaterials. However, currently available CA15-3 immunosensors still suffer from several limitations, such as relatively narrow linear dynamic ranges, dependence on optical or photoelectronic components, and insufficient evidence of long-term stability and reproducibility in complex serum matrices. To address these issues, we designed a new CA15-3 electrochemical immunosensor based on a novel combination of functional materials, aiming to achieve a wider working range, a lower detection limit, and improved stability. This strategy is expected to provide a useful approach for the early diagnosis and prevention of breast cancer.

“Metal–organic frameworks” (MOFs) are well-ordered microporous crystalline frameworks constructed by the spontaneous assembly of organic ligands and transition metal ions through coordination bond [15]. Due to their higher surface area (SA), adjustable pore structure and good physicochemical stability, MOFs are often exploited for electronic and sensing applications [16]. The stability of MOFs is a function of the strength of the coordination bonds; high-valent metal ions with higher charge densities can have reinforced coordination linkages with the carboxylate ligands, which in turn strengthens the framework stability. However, most MOFs have poor electrical conductivity, restricting their further applications [17,18]. In addition, their large porosity can decrease selectivity for molecules of a certain size [19]. To address these limitations, researchers have embedded conductive nanomaterials, including AuNPs, carbon nanotubes, and graphene, into the MOF structures to enhance their electrical performance, durability, and permeability [20,21].

Among different MOFs, ZIF-8 is a well-known structure, comprising 2-methylimidazole (2-Mim) coordinated with zinc [22]. ZIF-8 has a zeolite-like topology with a high SA and high porosity. In biosensing applications, the porous structure of ZIF-8 enables efficient loading of nanoparticles, enzymes, antibodies, or DNA probes. Its large specific surface area provides abundant sites for the binding of nanoparticles and biomolecules, while its pore channels can regulate the transport of nanoparticles and their substrate molecules, thereby improving the selectivity of the sensor [23]. As a result, ZIF-8 is a very useful platform for building highly sensitive electrochemical biosensors [24].

rGO is a graphene derivative achieved by eradicating most of the “oxygen-containing groups” by chemical, physical or thermal reduction. It has high SA, good electrical conductivity, adjustable surface chemistry and good mechanical stability. Additionally, it is therefore an ideal material for electrochemical sensors [25]. Its two-dimensional lamellar structure offers a myriad of active sites for the immobilization of biomolecules such as enzymes, antibodies, and nucleic acid aptamers. Moreover, the excellent conductivity of rGO facilitates the fast electron transfer (ET) at the electrode interface, which enhances the sensor sensitivity. The reduction reaction or surface functionalization can be optimized to improve the biomolecule interaction efficiency [26]. In order to further boost the sensor’s performance, the rGO was combined with AuNPs to prevent the aggregation of NPs, increase the electrocatalytic activity, and provide stable immobilization sites. This synergy has enabled the advancement of high-performance electrochemical biosensors to identify disease biomarkers, environmental pollutants, and pathogenic microorganisms.

AuNPs as a type of noble metal nanomaterials have been widely used in biosensors due to their intense localized surface plasmon resonance (LSPR), high conductivity, biocompatibility and tunable surface chemistry [27]. Their high specific SA and high surface energy provide multiple binding sites for the immobilization of biorecognition elements such as antibodies, enzymes, and nucleic acids, without loss of their biological concentration. By optimizing synthesis conditions, such as the use of reducing agents, stabilizers, and reaction conditions, the size, shape, and surface chemistry of AuNPs can be tightly controlled, improving their interaction with biomolecules and suppressing aggregation [28]. In recent years, these properties have been broadly used to enhance the performances of electrochemical biosensors for specific molecular detection.

In our design, the three nanomaterials play complementary roles within a hierarchical CS@AuNPs/ZIF-8/rGO interface. The ZIF-8/rGO underlayer provides a nanostructured scaffold with large surface area and porous channels, which increases the effective interfacial area and facilitates mass transport of the redox probe and analyte. On top of this scaffold, the CS@AuNPs layer acts as the main biofunctional film: AuNPs offer strong affinity toward the functional groups of antibodies, while the CS matrix ensures good biocompatibility and stabilizes the immobilized proteins. rGO throughout the composite forms a conductive network that bridges the nanostructures and accelerates electron transfer. Thus, rather than serving as the direct anchoring site for antibodies, ZIF-8 primarily enhances the loading capacity and interfacial architecture, while CS@AuNPs provide dense, stable binding sites and signal amplification. This synergistic combination allows the sensor to achieve high antibody loading, efficient charge transfer and improved analytical performance.

## 2. Materials and Methods

### 2.1. Chemicals and Reagents

The CA15-3 antigen, CA15-3 antibody and CYFRA21-1 antigen were purchased from “Beijing Keyue Zhongkai Technology Co., Ltd.” rGO was sourced from “Xianfeng Nanomaterials Technology Co., Ltd.(Jiangsu, China).” “Zinc nitrate hexahydrate” (Zn(NO_3_)_2_·6H_2_O) and 2-Mim (C_4_H_6_N_2_) were obtained from “Aladdin Reagent Co., Ltd. (Shanghai, China).” Hydrochloroauric acid tetrahydrate (HAuCl_4_·3H_2_O) and N,N-dimethylmethanamide purchased from Shanghai Macklin Biochemical Co., Ltd. (Shanghai, China). Chitosan (CS, average molecular weight ≈ 50,000 Da; Macklin Biochemical Co., Ltd. (Shanghai, China). Analytical-grade sulfuric acid, methanol, ethanol, acetic acid, potassium ferric cyanide, potassium ferro cyanide, and potassium chloride (KCL) were bought from “Guoyao Group Chemical Reagent Co., Ltd. (Shanghai, China).” BSA, ascorbic acid (AA), glucose (Glu), and uric acid (UA) were bought from “Sigma-Aldrich” (St. Louis, MO, USA). 0.01 M Phosphate-Buffered Saline (PBS) (pH = 7.4) with 5 mmol/L [Fe(CN)_6_]^3−/4−^ and 0.1 M KCL was bought from Feijin Biotechnology Co., Ltd. (Fuzhou, China).

### 2.2. Instrumentation

EDS (Energy-Dispersive X-ray Spectroscopy), SEM (Scanning Electron Microscopy), and TEM (Transmission Electron Microscopy) were carried out with Talos F2001 instrument (Thermo Fisher Scientific, New York, NY, USA). FT-IR (Fourier Transform Infrared Spectroscopy) spectra were observed by an “IRTracer-100 spectrometer” (Shimadzu, Japan). XRD (X-ray Diffraction) were performed on a “D8 Advance diffractometer” (Bruker, Germany) and XPS (X-ray Photoelectron Spectroscopy) measurements were performed on an “ESCALAB 250 instrument” (Thermo Fisher Scientific, USA).

Electrochemical experiments were performed with a “CS150H single-channel electrochemical workstation” (Kost Instruments Co., Ltd. Wuhan, China). A “three-electrode system” was utilized, including a saturated Ag/AgCl electrode, platinum wire, and CS@AuNPs/ZIF-8/rGO-modified glassy carbon electrode (GCE) utilized as the reference, counter, and working electrodes, respectively. The GCE had a cross-sectional diameter of 3 mm.

Other laboratory equipment was a vacuum oven (Hecheng Instrument & Equipment Co., Ltd. (Shanghai, China), “DHG-9030A constant-temperature blast oven” (Nuhang Instrument & Equipment Co., Ltd. Shanghai, China), SN-QX-32D ultrasonic cleaner (Shangpu Instrument & Equipment Co., Ltd. Shanghai, China), and FB224L analytical balance with internal calibration (Shunyu Hengping Science & Technology Instrument Co., Ltd., Shanghai, China). Ultrapure water was supplied by the Wuhan Pinguan ultrapure water system (Pinguan Instrument Co., Ltd. Wuhan, China) with resistivity of 18.25 MΩ∙cm.

### 2.3. Fabrication of ZIF-8 and ZIF-8/rGO Composite Materials

ZIF-8 was synthesized in an aqueous system. Briefly, 0.744 g of Zn(NO_3_)_2_·6H_2_O and 12.3 g of 2-methylimidazole were separately dissolved in 10 mL and 90 mL of double-distilled water (DDW), respectively. The two solutions were then mixed and stirred for 24 h. The resulting precipitate was collected by centrifugation at 4000 rpm for 40 min and washed three times with 10 mL of DDW to remove any residual precursors. Finally, the sample was dried under vacuum at 60 °C for 48 h to obtain ZIF-8.

The ZIF-8/rGO nanocomposites were made by an in situ growth method with rGO as a structural template [29]. Specifically, 0.041 g and 0.074 g of Zn(NO_3_)_2_·6H_2_O and 2-Mim, respectively, were dissolved in two separate 10 mL portions of methanol. Afterwards, 10 mg of rGO was dispersed in Zn(NO_3_)_2_·6H_2_O methanolic solution and then exposed to ultrasonic energy below 30 °C for 6 h to achieve homogeneous dispersion. Thereafter, the solution of 2-Mim was poured to the above mixture and stirred for 6 h to promote the growth of the ZIF-8 crystals on rGO sheets.

The obtained suspension was centrifuged (40 min, 4000 rpm) to obtain the precipitate that was rinsed 3 times using 10 mL of methanol to remove residual precursors. Lastly, the purified product was vacuum-dried at 60 °C for 4 h to obtain ZIF-8/rGO nanocomposite.

### 2.4. Fabrication of CS@AuNPs Composite

CS@AuNPs were produced using the decrease in chloroauric acid trihydrate (HAuCl_4_·3H_2_O) in the presence of CS as the stabilizing and reducing agent [30]. Specifically, CS (50 mg) was dissolved in acetic acid solution (1% *v*/*v*, 10 mL) and stirred continuously until a clear solution was achieved. After that, 20 µL of 0.1 M HAuCl_4_·3H_2_O was added slowly dropwise under stirring. The solution’s color slowly changed from pale yellow to purplish red, which indicated the successful AuNPs’ formation. The obtained CS@AuNPs were preserved at 4 °C for further use.

### 2.5. Construction of Immunosensors and Electrochemical Detection

The GCE was first polished with 0.3 um and 0.05 um alumina slurry on chamois leather until a mirror-like surface was obtained. The electrode was then ultrasonically cleaned nitric acid (50% *v*/*v*) for 5 min successively, pure ethanol and distilled water. After cleaning, the electrode was electrochemically activated in 3% H_2_SO_4_ by potential cycling: dissolution/deposition at 2.0 V, for 60 s, followed by 0.9 V for 30 s, and “cyclic voltammetry” (CV) scanning from −0.9 V to 1.1 V at a scan rate of 100 mV/s. After that, 3 µL of the ZIF-8/rGO dispersion (2 mg/mL) was drop cast on the GCE surface and dried below 39 °C. Then, CS@AuNPs solution (5 µL) was applied on the electrode surface (ES) and dried at 39 °C to form a stable film. Then 5 µL of CA15-3 antibody solution (50 µg/mL) was applied to the ES and incubated at 4 °C for the entire night. In order to block the nonspecific binding sites, the electrode was incubated in 1% (*v*/*v*) BSA solution for 30 min at 25 °C, and then rinsed with PBS and dried at 37 °C. After each modification and drying step, the electrode was rinsed with PBS to eradicate the unbound material.

The standard solution of CA15-3 (0.001, 0.01, 0.1, 0.10, 0.100, 0.200 and 0.400 U/mL) was prepared by gradient dilution method in PBS and stored at 4 °C. The modified electrode was then covered with 5 µL of antigen solution of CA15-3 added dropwise from lower to higher activity. After incubation for 30 min at 37 °C, “differential pulse voltammetry” (DPV) measurements were performed in an electrolyte containing 0.01 mol/L PBS (pH = 7.4), 5 mM [Fe(CN)_6_]^3−/4−^, and 0.1 M KCl. The DPV parameters were voltage amplitude; 0.06 V, pulse duration; 0.08 s, pulse period; 0.3 s, start voltage; 0.05 V, end voltage; 0.45 V, and voltage step; 0.002 V. The maximum current signal recorded in the absence of CA15-3 antigen was taken as the blank signal (I_0_). The corresponding current (I_P_) was recorded after incubation with different contents of the antigen. The “change in current” (ΔI = I_0_ − I_P_) was used to measure the activity of the CA15-3, and higher antigen activities caused a larger decrease in peak current because of an antigen–antibody complex’s formation at the electrode interface.

In this study, the limit of detection (LOD) was calculated as LOD = 3σ/s, where σ is the standard deviation of the blank signal, s is the slope of the linear regression equation for the actual sample. The relative standard deviation (RSD, %) was calculated using the following formula: RSD (%) = SD/M × 100%, where SD is the standard deviation and M is the mean value. All statistical analyses were performed at a 95% confidence level.

### 2.6. Collection and Preparation of Serum Samples

Serum samples from BC patients (*n* = 10) and healthy subjects (*n* = 10) were collected from “Tianmen First People’s Hospital, Wuhan University of Science and Technology.” The study was approved by the “hospital ethics committee” and informed consent was received from all the study subjects before sample collection. To remove interference by other proteins, the collected blood samples were centrifuged (15 min, 4000 rpm). The serum was isolated carefully and stored at −20 °C for further analysis.

## 3. Results and Discussion

### 3.1. Operating Principle and Procedure of Immunosensor

**Figure 1:**
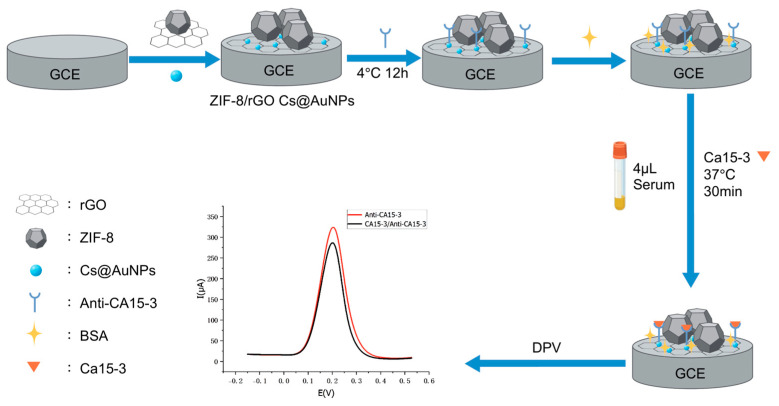
Schematic diagram of testing procedure and electrode preparation.

In Figure 1, an E-IS was fabricated via the CS@AuNPs/ZIF-8/rGO composite as the modification material. ZIF-8 and rGO were drop-cast onto the GCE surface as a mixed suspension. The rGO sheets formed a two-dimensional supporting framework on the electrode, while ZIF-8 crystals preferentially anchored onto this framework, increasing the interparticle spacing and preventing the formation of large aggregates. During the synthesis of CS@AuNPs, chitosan coated and stabilized the nanoparticle surfaces. The ZIF-8/rGO layer thus served as the first underlying scaffold, whose relatively rough, high-surface-area interface enabled the second CS@AuNPs layer to spread more uniformly over the electrode, reducing local accumulation compared with a smooth bare GCE. In addition, sulfhydryl (–SH) groups on the anti-CA15-3 antibodies can bind to AuNPs via Au–S interactions, allowing stable and efficient antibody immobilization.

The biomacromolecules CA15-3 antibody, BSA, and CA15-3 antigen when sequentially assembled on the electrode surface (ES) inhibit the electron-transfer (ET) of electrochemical probe ions (e.g., [Fe(CN)_6_]^3−/4−^). This leads to a gradual decline in the peak current obtained by DPV. Upon introduction of the antigen, the specific antigen–antibody interaction forms immunocomplexes which further increase steric hindrance and further inhibit ET at the electrode interface. The more the antigen activity, the more the immunocomplexes formed, the more the obstruction to ET and the more the corresponding current difference (ΔI).

Therefore, the activity of the tumor marker CA15-3 in human serum can be quantitatively measured by accurately measuring the change in the DPV peak current (ΔI). This method offers a sensitive and reliable analytical method for the clinical diagnosis and early screening of BC.

### 3.2. Morphological and Structural Characteristics of Materials

#### 3.2.1. SEM Morphological Characterization

**Figure 2:**
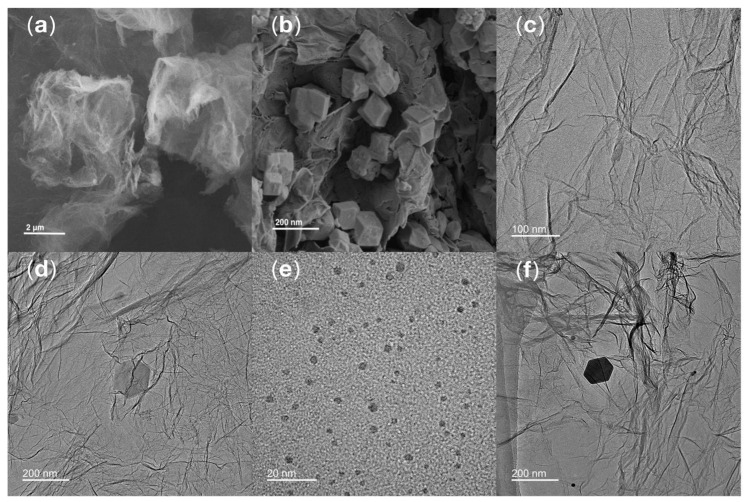
SEM and TEM images of the materials: (**a**) SEM of rGO; (**b**) SEM of ZIF-8/rGO; (**c**) TEM of rGO; (**d**) TEM of ZIF-8/rGO; (**e**) TEM of CS@AuNPs; (**f**) TEM of CS@AuNPs/ZIF-8/rGO.

SEM was applied to analyze the structural features of rGO and synthesized ZIF-8/rGO composite. In Figure 2a, the SEM image of rGO shows a typical 2-dimensional layered wrinkled morphology of gauze-like structure, which is typical of rGO. In Figure 2b, the uniformly anchored regular dodecahedral ZIF-8 nanocrystals on the wrinkled rGO sheets are observed. The results show that some ZIF-8 crystals are embedded in the interlayers of rGO and some are adsorbed closely on its surface. Together they constitute a compact and well-integrated heterostructure, which clearly demonstrates the successful composite formation between ZIF-8 and rGO. Compared with ZIF-8 or rGO used alone, this architecture combines the high porosity of ZIF-8 with the conductive framework of rGO, which is expected to alleviate the aggregation of ZIF-8 and provide more efficient pathways for electron transport.

#### 3.2.2. TEM Morphological Characterization

The morphology of rGO, CS@AuNPs, ZIF-8/rGO and the final composite CS@AuNPs/ZIF-8/rGO was explored by TEM. As displayed Figure 2c, the TEM image of rGO shows an ultrathin, translucent, and film-like structure with well-defined edges. The rGO sheets have typical curled edges which overlap and intertwine to form a layered morphology. Figure 2d shows the TEM image of ZIF-8/rGO, and it can be observed that ZIF-8 crystals are uniformly affixed onto the rGO surface. The particles show a regular rhombic dodecahedral shape typical of well-formed ZIF-8 crystals. The TEM morphology of CS@AuNPs as shown in Figure 2e shows that the AuNPs are evenly dispersed in the CS matrix and have a close to spherical shape. Figure 2f indicates the TEM image of the final composite of CS@AuNPs/ZIF-8/rGO, in which CS@AuNPs are homogeneously distributed on the ZIF-8 particles. These observations confirm the effective synthesis of the CS@AuNPs/ZIF-8/rGO hybrid material with well-integrated structural features from all individual components. Compared with the CS@AuNPs or the individual ZIF-8/rGO system, the composite material further enlarges the electrochemically active surface area and provides well-dispersed sites for antibody immobilization. The Supplementary Figure S1 regarding TEM can be found in the Supporting Information Section.

**Figure 3:**
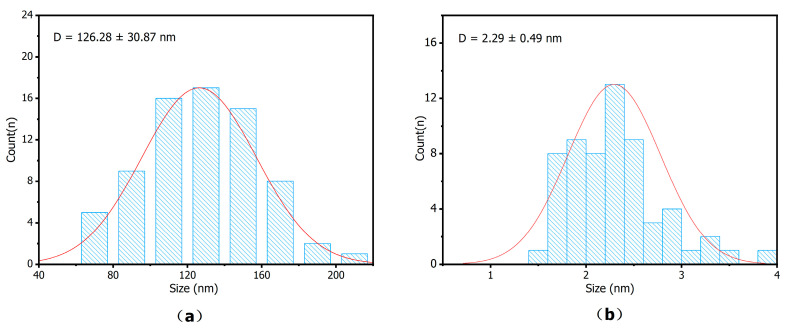
Particle size distribution histogram of the materials: (**a**) ZIF-8; (**b**) CS@AuNPs.

The particle size of ZIF-8 was statistically analyzed from the SEM images, and that of AuNPs was analyzed from the TEM images. As shown in Figure 3, the diameter of ZIF-8 is 126.28 ± 30.87 nm, and the diameter of CS@AuNPs is 2.29 ± 0.49 nm.

#### 3.2.3. EDS Characterization

**Figure 4:**
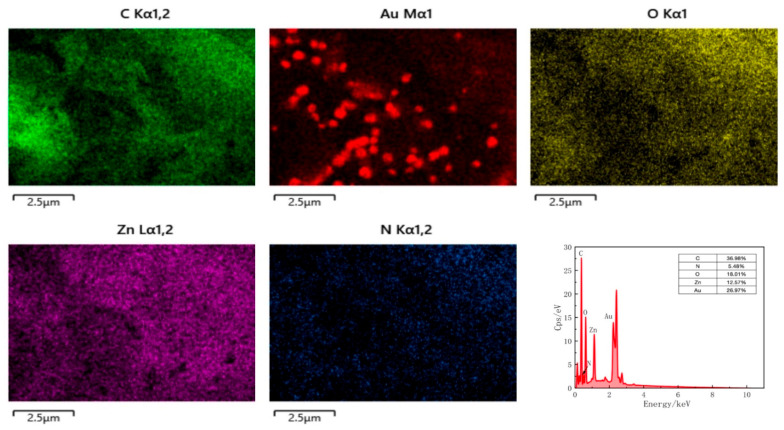
EDS mass distribution, elemental distribution, and surface scan of CS@AuNPs/ZIF-8/GO.

In addition, to verify the successful fabrication of the composite, the elemental distribution of the CS@AuNPs/ZIF-8/rGO material was characterized by EDS (Figure 4). The figure indicates the morphology of the CS@AuNPs/ZIF-8/rGO composite and elemental mapping of C, N, O, Zn, and Au, respectively.

The EDS analysis showed that the composite is composed of C (36.98%), N (5.48%), O (18.01%), Zn (12.57%), and Au (26.97%). The components of C and O are mainly from rGO, whereas N and Zn are from ZIF-8 framework. The obvious presence of Au indicates the effective deposition of AuNPs onto the ZIF-8/rGO substrate. Furthermore, the homogeneous distribution of those elements confirms the effective synthesis and homogeneous composition of the CS@AuNPs/ZIF-8/rGO hybrid material.

#### 3.2.4. FT-IR Characterization

**Figure 5:**
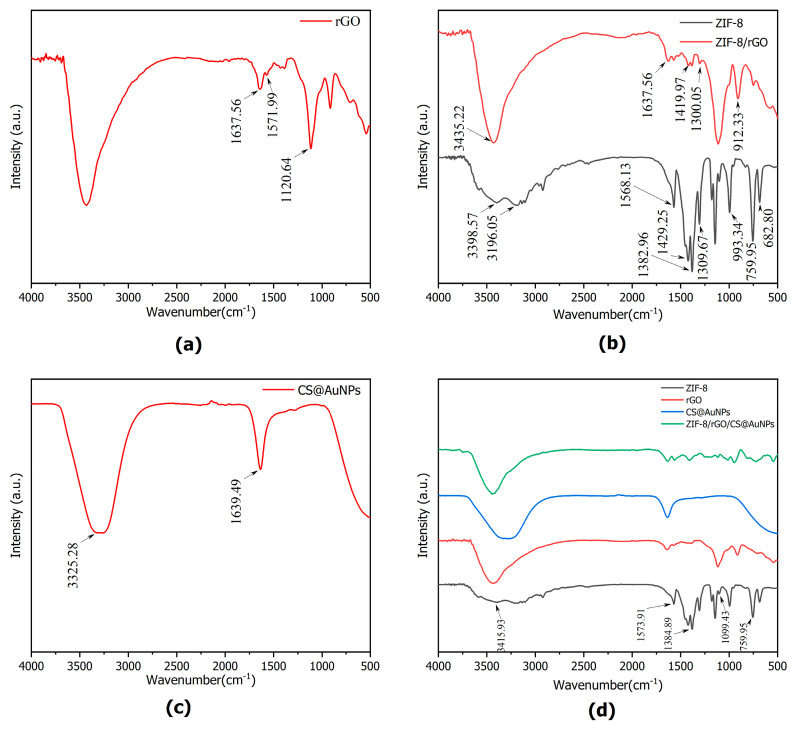
FT-IR images of the materials: (**a**) rGO; (**b**) ZIF-8, ZIF-8/rGO; (**c**) CS@AuNPs; (**d**) ZIF-8, rGO, CS@AuNPs,CS@AuNPs/ZIF-8/rGO.

The structural features of the prepared materials were characterized by FT-IR spectroscopy. In Figure 5a, the rGO’s FT-IR spectrum shows an intense and broad absorption band at 3420 cm^−1^, which is due to the stretching vibrations (ν) of the residual hydroxyl (-OH) groups. This band is most probably due to incompletely reduced oxygen-containing groups or adsorbed water molecules. The strong peak at 1637 cm^−1^ is allotted to the ν(C=C) of the sp^2^-hybridized carbon backbone, which is a typical feature of graphene.

As shown in Figure 5b, the non-template synthesized ZIF-8 exhibits two absorption peaks at 3398 cm^−1^ and 3196 cm^−1^, attributed to N-H/O-H stretching vibrations associated with the 2-methylimidazole ligand and adsorbed water molecules. In the ZIF-8/rGO composite, these high-wavenumber features are significantly attenuated and merge with the O–H/C–H stretching vibrations of rGO, resulting in a broad peak centered around 3435 cm^−1^. In the fingerprint region of 1600–1300 cm^−1^, ZIF-8 shows several characteristic peaks at approximately 1568, 1429, 1383, and 1309 cm^−1^, corresponding to C=N and C-N stretching vibrations of the imidazole ring and C–H in-plane bending vibrations. These characteristic peaks remain clearly observable in the ZIF-8/rGO spectrum at 1637, 1420, and 1309 cm^−1^, which are absent in the rGO spectrum, confirming that the ZIF-8 framework structure is maintained on the rGO sheets. Compared with pristine ZIF-8, the slight peak shifts and intensity changes of these bands in the composite suggest interactions between the ZIF-8 coordination environment and the oxygen-containing functional groups as well as the π-conjugated surface of rGO.

Figure 5c shows the CS@AuNPs’ FT-IR spectrum. The strong absorption peak at 3325 cm^−1^ can be allotted to the ν(N-H) and ν(O-H) of CS, and the absorption peak at 1639 cm^−1^ can be allocated to the amide I ν(C=O).

Figure 5d shows the FT-IR spectra of the three separate components and the final CS@AuNPs/ZIF-8/rGO composite, which combines the characteristic peaks of all components. The blue-shifted C=N vibration at 1595 cm^−1^ from ZIF-8/rGO composite is still visible and the amide I peak at 1639 cm^−1^ typical of CS is also clearly observed. These spectra provide indication for the effective incorporation of CS@AuNPs with ZIF-8/rGO hybrid to generate a ternary composite structure.

#### 3.2.5. XRD Characterization

**Figure 6:**
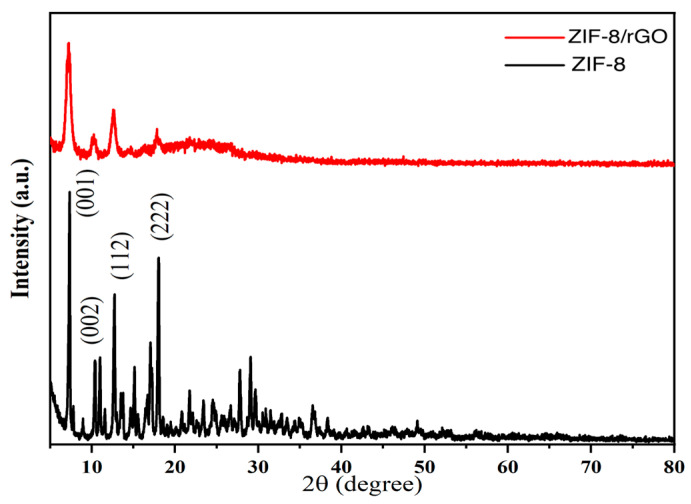
XRD Patterns of ZIF-8 and ZIF-8/rGO.

Figure 6 illustrates the characteristic diffraction peaks of ZIF-8 at crystal plane positions (001), (112), (222) and (002) with multiple reflections appearing in the 10–80° range. These peaks are consistent with the usual polycrystalline diffraction features of ZIF-8. The sharp and well-defined peaks demonstrate the high crystallinity of ZIF-8 and their positions and intensities are very similar to the standard ZIF-8 diffraction pattern. In ZIF-8/rGO composite, the characteristic diffraction peaks of ZIF-8 are clearly observed without any significant broadening or shift, suggesting that the introduction of rGO does not affect the crystalline framework of ZIF-8. Additionally, a broad peak obtained at about 23.5° corresponding to the (002) plane of rGO is the typical structural feature of rGO. The coexistence of this broad rGO peak and the sharp ZIF-8 peaks suggests the successful incorporation of rGO into the composite without affecting the original crystalline integrity of ZIF-8. Furthermore, the existence of both the diffuse (002) peak of rGO and the well-defined diffraction peaks of ZIF-8 at the same time is indicative of a strong physical interlocking between the two components, rather than the formation of a chemical bond. This structural integration improves the electrical conductivity and structural integrity of the composite and offers other active interfaces that are useful for electrochemical and catalytic applications.

#### 3.2.6. XPS Characterization

**Figure 7:**
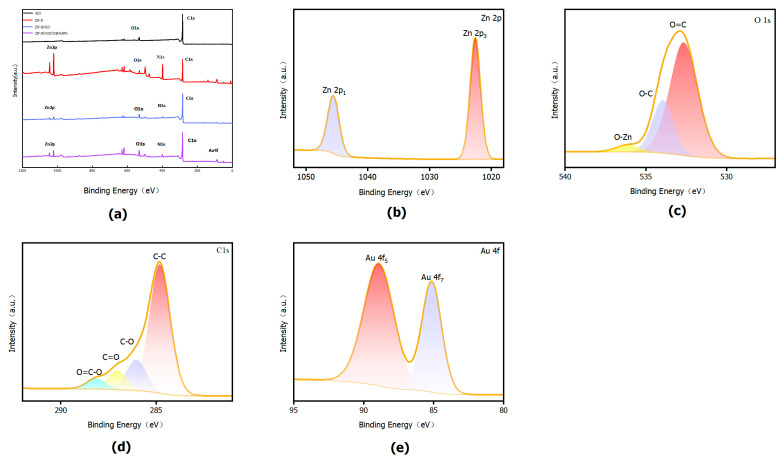
XPS images of the materials: (**a**) XPS survey spectra of rGO, ZIF-8, ZIF-8/rGO and rGO/ZIF-8/CS@AuNPs; (**b**) High-resolution Zn 2p XPS spectrum of rGO/ZIF-8/CS@AuNPs; (**c**) High-resolution O 1s XPS spectrum of rG-O/ZIF-8/CS@AuNPs; (**d**) High-resolution C 1s XPS spectrum of rGO/ZIF-8/CS@AuNPs; (**e**) High-resolution Au 4f XPS spectrum of rGO/ZIF-8/CS@AuNPs.

As shown in Figure 7, XPS was used to analyze the surface elemental composition and chemical states of the samples. The survey spectrum of rGO is dominated by C 1s with a minor O 1s signal, indicating that only a small amount of oxygen-containing functional groups remains on its surface. In contrast, pure ZIF-8 exhibits pronounced Zn 2p and N 1s peaks, accompanied by changes in the relative intensities of C 1s and O 1s, reflecting the presence of organic ligands and Zn–N coordination nodes. Compared with rGO, the ZIF-8/rGO composite shows markedly enhanced N 1s and Zn 2p signals and a reduced relative contribution of C 1s, suggesting that ZIF-8 is grown as crystalline domains on the carbon support rather than being simply physically mixed. After introducing CS@AuNPs, the N 1s and O 1s peaks of rGO/ZIF-8/CS@AuNPs further increase, and additional Au 4f signals appear in the 80–90 eV region, confirming the successful incorporation of amino/hydroxyl-containing chitosan and Au nanoparticles into the system. These features are favorable for generating more coordination-active sites and polar adsorption centers.

In the high-resolution Zn 2p spectrum of rGO/ZIF-8/CS@AuNPs, a typical spin–orbit doublet is observed at approximately 1021 eV (Zn 2p_3/2_) and 1044 eV (Zn 2p_1/2_), with an energy separation of about 23 eV, consistent with Zn^2+^ in ZIF-8. Compared with pure ZIF-8, the Zn 2p peaks in the composite are slightly shifted to higher binding energy, indicating a slight decrease in the electron density of Zn^2+^ due to interactions with oxygen- and nitrogen-containing groups on rGO and chitosan. This suggests that, while the ZIF-8 framework remains intact, its local electronic environment is modulated by the support.

The O 1s high-resolution spectra can be deconvoluted into three main components corresponding to O–Zn, C–O/C–O–C, and carbonyl/carboxyl O=C species. In the composite, the latter two components increase significantly relative to pure ZIF-8, reflecting the higher density of oxygen-containing functional groups introduced by rGO and chitosan. In the C 1s spectra, besides the main C–C/C=C peak, the contributions of polar carbon species such as C–O and O–C=O are enhanced in ZIF-8/rGO and rGO/ZIF-8/CS@AuNPs, further confirming that the organic ligands of ZIF-8 and chitosan chains are successfully anchored on the graphene surface, providing more hydrophilic and coordination sites.

For rGO/ZIF-8/CS@AuNPs, the high-resolution Au 4f spectrum shows a clear doublet at approximately 84–85 eV (Au 4f_7/2_) and 88–89 eV (Au 4f_5/2_), indicating electronic interactions between Au and the chitosan ligands, which render Au partially positively charged. This confirms that the Au nanoparticles are effectively loaded on the surface of the composite. Overall, the XPS results demonstrate, from both elemental composition and electronic structure, the coexistence and strong coupling of rGO, ZIF-8, CS, and AuNPs in the rGO/ZIF-8/CS@AuNPs system.

### 3.3. Electrochemical Performance Evaluation of Immunosensor

**Figure 8:**
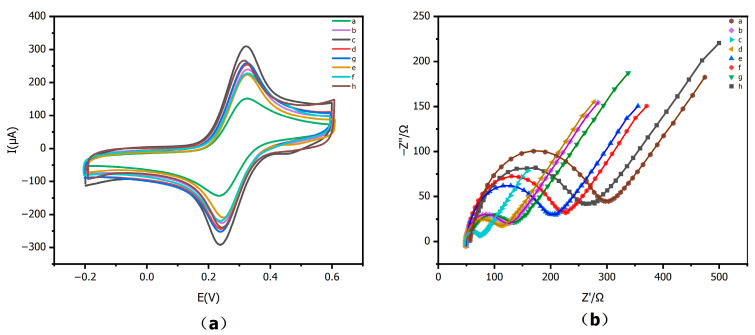
Electrochemical characterization: (**a**) CV; (**b**) EIS. Curves a–h: a.Bare GCE, b.ZIF-8/rGO/GCE, c.CS@AuNPs/ZIF-8/rGO/GCE, d.Anti-CA15-3/CS@AuNPs/ZIF-8/rGO/GCE, e.BSA-Anti-CA15-3/CS@AuNPs/ZIF-8/rGO/GCE, f.CA15-3/BSA-Anti-CA15-3/CS@AuNPs/ZIF-8/rGO/GCE, g.CS@AuNPs/GCE, h.rGO/GCE.

To validate the performance of the immunosensor, CV and EIS measurements were carried out in an electrolyte containing 5 mM [Fe(CN)_6_]^3−/4−^ and 0.1 M KCl (pH 7.4). Figure 8a,b show the CV curves and EIS plots of the electrodes at different modification stages. As shown in Figure 8a, the bare GCE (curve a) exhibits the smallest redox peak current. CS@AuNPs (curve g) and rGO (curve h) are the main contributors to enhancing the interfacial conductivity. In ZIF-8/rGO (curve b), ZIF-8 indirectly improves the loading of nanoparticles by providing a high specific surface area and a nanostructured framework. When the first ZIF-8/rGO layer (curve b) is further coated with a second CS@AuNPs layer (curve g) to construct the bilayer CS@AuNPs/ZIF-8/rGO structure (curve c), the peak current increases markedly, indicating that the bilayer configuration generates a stronger electrochemical response than the corresponding single-layer structures. This can be attributed to the fact that the bottom ZIF-8/rGO layer offers a high surface area and a continuous conductive network, supplying more exposed active sites and low-resistance pathways for electron transfer between the electrode and the electrolyte interface. The top CS@AuNPs layer introduces a large number of uniformly dispersed catalytic sites, while the chitosan film helps to stabilize the nanoparticles and improve interfacial contact with the electrode surface. As a result, the electrode modified with the bilayer material delivers higher peak currents and superior electrochemical performance. Upon successive immobilization of CA15-3 antibody, antigen, and BSA on the CS@AuNPs/ZIF-8/rGO/GCE (curves d, e, and f), the peak currents decrease stepwise, because the formation of an insulating protein layer at the electrode surface hinders the access of the [Fe(CN)_6_]^3−/4−^ redox probe to the interface and increases the resistance to electron transfer.

This interpretation is further supported by the EIS results shown in Figure 8b. According to the Randles equivalent circuit model, the diameter of the semicircle in the high-frequency region is associated with the charge-transfer resistance (Rct). The semicircle corresponding to the CS@AuNPs/ZIF-8/rGO-modified electrode is smaller than those of the bare GCE and the single-layer ZIF-8/rGO electrode, indicating a lower R_ct and faster interfacial electron transfer. The subsequent increase in R_ct after immobilization of the biomolecular layers is consistent with the formation of a nonconductive film. The good agreement between the CV and EIS data demonstrates that, compared with single-layer ZIF-8/rGO (curve b) or CS@AuNPs (curve g), the bilayer CS@AuNPs/ZIF-8/rGO structure (curve c) is more effective in reducing Rct and facilitating electron transfer. The observed current responses are in line with the properties of the electrode modification materials, confirming the successful construction of the electrochemical immunosensor.

### 3.4. Optimization of Experimental Conditions

The loading density of the nanomaterials, the amount of antigen applied, and the incubation time all have a marked influence on the analytical performance of the immunosensor. Optimization of the loading densities of ZIF-8/rGO and CS@AuNPs can enlarge the electrochemically active surface area, provide more active sites, and accelerate substrate diffusion, while avoiding excessively thick films that would lead to surface saturation. In addition, the amount of CA15-3 antigen deposited, and the incubation time affect antibody binding, nonspecific adsorption, and protein stability. For these reasons, these four experimental parameters were selected for systematic optimization and discussion.

The change in current is defined as ΔI = I_0_ − I_P_, where I_0_ is the peak current before the addition of CA15-3 and IP is the peak current after incubation with CA15-3. The mass loading density of the electrode surface, L (mg/cm^2^), is calculated as L = C × V/A, where C is the concentration of the corresponding solution (mg/mL), and A is the geometric area of the 3 mm diameter GCE (A ≈ 0.071 cm^2^).

**Figure 9:**
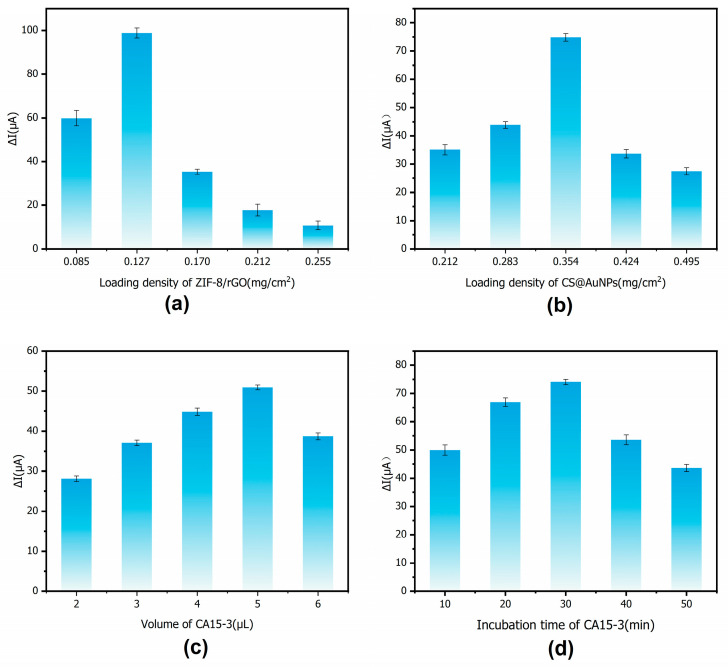
Condition optimization: (**a**) Loading density of ZIF-8/rGO; (**b**) Loading density of CS@AuNPs; (**c**) Dispensing volume of CA15-3; (**d**) Incubation time of CA15-3 antigen.

In Figure 9a, the optimal loading of ZIF-8/rGO was determined by varying its mass loading density (0.085, 0.127, 0.170, 0.212, and 0.255 mg/cm^2^) while keeping other conditions constant. The maximum current response was obtained at a loading density of 0.127 mg/cm^2^. As the ZIF-8/rGO loading density increases, a continuous conductive network is formed on the electrode surface and a larger specific surface area is provided, which enlarges the active area and reduces the charge-transfer resistance. When the loading density is further increased, however, the multilayer-stacked composite forms a thick and dense film on the electrode, extending the diffusion path of the redox probe, increasing tortuosity of the pores, and lowering the effective diffusion coefficient.

In Figure 9b, the optimal amount of CS@AuNPs was determined by varying its mass loading density (0.212, 0.283, 0.354, 0.424, and 0.495 mg/cm^2^). The best current response was obtained at a loading density of 0.345 mg/cm^2^. With increasing loading density, the good film-forming ability of chitosan and the high conductivity of AuNPs act synergistically: more hydrophilic functional groups become available for antibody immobilization, and the interfacial charge-transfer resistance is significantly reduced, leading to a gradual increase in peak current. When the loading density exceeds the optimal value, the chitosan film becomes excessively thick and AuNPs tend to aggregate, which decreases the effective surface area, blocks part of the pores, and thickens the diffusion layer. Mass transport of the redox probe is then hindered, offsetting the benefits of improved conductivity and causing the current response to decline.

In Figure 9c, dispensing volume of CA15-3 (2, 3, 4, 5, and 6 μL) was evaluated. The highest current response was observed at 5 μL. At low antigen dispensing volume, the amount of CA15-3 available for specific binding to the antibody is limited, and the interfacial immunocomplex layer is not fully formed, resulting in a relatively small current change. As the dispensing volume approaches the optimal value, the antigen amount becomes sufficient to occupy most of the available binding sites, and the current response reaches its maximum. Further increasing the antigen dispensing volume beyond this point does not provide additional effective binding sites; instead, excess antigen is more likely to be nonspecifically adsorbed or stack into multilayers, thickening the protein film, altering the local interfacial microenvironment and diffusion pathways, and thereby impeding mass transport of the probe. Consequently, the current signal decreases.

Figure 9d shows the effect of antigen incubation time on the sensor response, with incubation times of 10, 20, 30, 40, and 50 min examined under otherwise optimized conditions. An incubation time of 30 min was found to be optimal. At shorter incubation times, a considerable fraction of antigen molecules is still diffusion-limited and has not yet reached or bound to the electrode surface, so the immunolabel does not achieve saturated coverage. As the incubation time is extended to 30 min, more antigen molecules occupy the binding sites and the current change increases significantly. Further extension to 40–50 min leads to only marginal improvements in specific binding, while excessively long incubation may induce conformational changes or partial deactivation of CA15-3 and promote nonspecific adsorption of matrix components, ultimately compromising protein stability.

### 3.5. Linear Range and LOD

**Figure 10:**
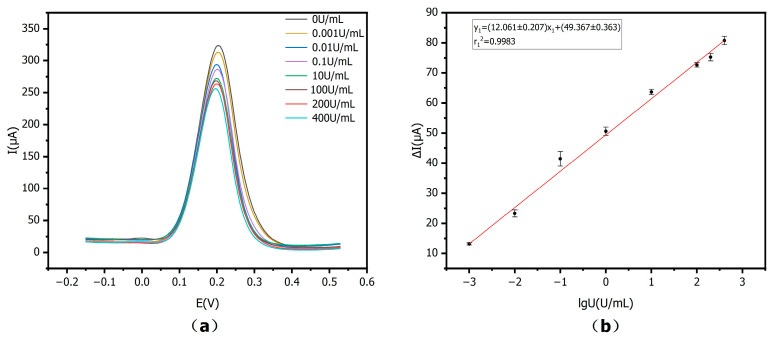
Standard calibration curve: (**a**) DPV values at different CA15-3 activities: 0, 0.001, 0.01, 0.1, 10, 100, 200, and 400 U/mL; (**b**) Linear equation for logarithm of CA15-3 activity versus peak current difference (*n* = 3).

DPV was used for measuring linear dynamic range and LOD for CA15-3 using the optimized experimental conditions mentioned above. As shown in Figure 10, by adding CA15-3 contents of 0–400 U/mL, Figure 10a shows the DPV response signals. In the absence of CA15-3, the sensor showed the highest signal of current. With increasing CA15-3 activities, the current signal gradually decreased. This decrease was due to the specific antigen–antibody binding, which caused the formation of immunocomplexes, which inhibited the ET at the ES, and thus the current response.

Figure 10b shows a strong linear correlation between the logarithm of antigen activity (lgU, U/mL) and the ΔI measured by DPV in 0–400 U/mL. In the revised manuscript, the X axis is defined as the logarithm of the CA15-3 antigen activity, lgU (U/mL), and the Y axis as the DPV current difference ΔI (µA) between the antibody-incubated and antigen-incubated electrodes. The calibration equation is now expressed as y_1_ = (12.061 ± 0.207) x_1_ + (49.367 ± 0.363), with r_1_^2^ = 0.9983, *n* = 3.

To assess the applicability of the E-IS in real samples, serum of healthy participants was utilized to prepare CA15-3 solutions. The serum was diluted with PBS in the dilution ratio from 1:100 to 1:1200. At lower dilution factors (such as 1:100), the current response for high CA15-3 activity tends to level off, indicating matrix-induced deviations from linearity, whereas at a 1:1000 dilution the signal varies more linearly and remains more stable. Therefore, a 1:1000 dilution is considered more suitable for the determination of CA15-3 in real serum samples in this work. The corresponding experimental results have been added as Table S1 in the Supporting Information. Therefore, a dilution ratio of 1:1000 was chosen as the best since it effectively minimized non-specific adsorption from serum components.

**Figure 11:**
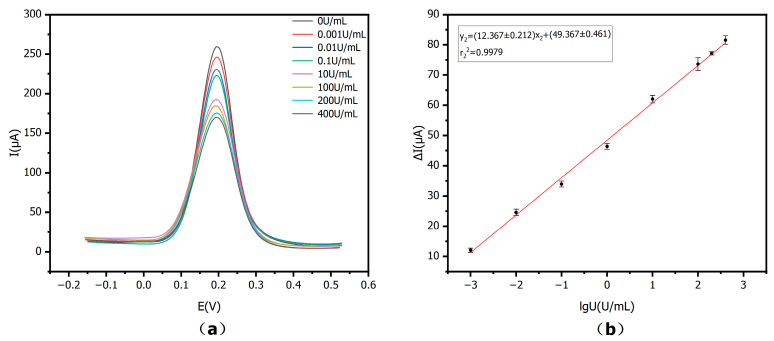
Matrix-matched calibration curve: (**a**) DPV values when incubating with various activities of CA15-3 prepared in normal human serum: 0, 0.001, 0.01, 0.1, 10, 100, 200, 400 U/mL; (**b**) Linear equation for logarithm of CA15-3 activity versus peak current difference (*n* = 3).

As revealed in Figure 11a, the ΔI showed a positive correlation with CA15-3 activity on a logarithmic scale in 0–400 U/mL. This linear relationship was similar to that obtained from the CA15-3 standard solutions prepared in PBS, indicating that the developed immunosensor retains reliable performance even in the more complex serum matrix. In Figure 11b, the matrix-matched calibration curve uses the same axis quantities and units as the standard curve, y_2_ = (12.367 ± 0.212) x_2_ + (49.367 ± 0.461), with r_2_^2^ = 0.9979, *n* = 3. The linear detection range was 0–400 U/mL. These results show that the proposed E-IS can be effectively utilized in CA15-3 detection in real serum samples with an accurate and wide detection range.

### 3.6. Sensor-Related Performance

Precision, which measures the stability of repeated measurements under the same conditions, is an important measure of sensor performance. In practical applications, a low relative standard deviation (RSD) (usually less than 10%) is an indication of stable and reliable detection results that are consistent with the requirements for measurement consistency and reliability. To assess the accuracy of the proposed E-IS, precision evaluation experiments were carried out under the optimal experimental conditions.

**Figure 12:**
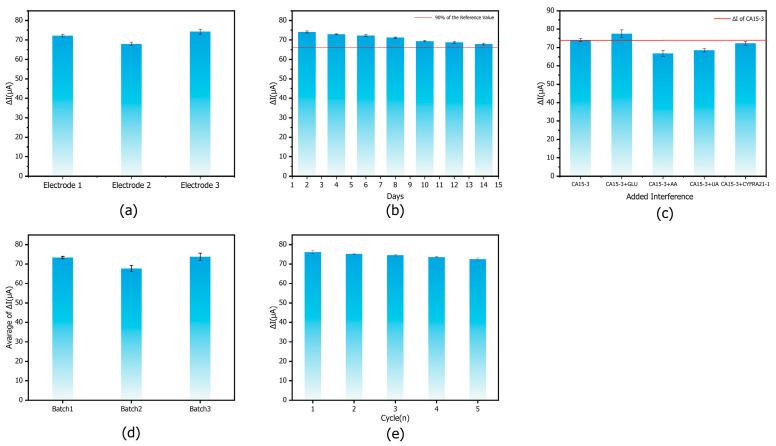
Sensor-related performance: (**a**) Accuracy of E-ISs; (**b**) Stability of E-ISs, the red line represents the 90% stability threshold of the initial signal of the immunosensor. (**c**) Selectivity of E-ISs, the red line represents the signal obtained when the immunosensor is incubated with CA15-3 only; (**d**) Batch-to-batch variation in E-ISs; (**e**) Reusability of E-ISs.

To evaluate the long-term stability of the E-Is, a treated glassy carbon electrode was incubated with 100 U/mL CA15-3 antigen and subjected to three consecutive DPV measurements. Thereafter, three measurements were repeated every 2 days. After each measurement, the electrode was rinsed with PBS, dried at 37 °C, and stored at 4 °C until the next test. As shown in Figure 12b, the E-Is retained approximately 90% of its initial signal over 14 days, indicating good long-term stability.

When detecting CA15-3 in human serum, other serum components may potentially interfere with the assay. To assess the selectivity of the E-IS in a complex serum matrix, Glu, AA, UA, and another tumor marker, CYFRA21-1, were selected as typical potential interferents. Figure 12c shows the changes in DPV peak current for a solution containing only CA15-3 and for solutions in which CA15-3 coexisted with each interferent. The activities of CA15-3 was 1 U/mL, and the activity of each coexisting species was 100 ng/mL. The peak currents exhibited only slight variations, with a relative standard deviation (RSD) of 5.83%, which is within an acceptable range. These results indicate that even at a significantly higher activity, these substances are not readily recognized or specifically bound by the E-IS. Therefore, the E-IS shows high selectivity toward CA15-3 and is suitable for reliable detection of CA15-3 in serum samples.

The batch-to-batch variability of the E-IS was examined using electrodes prepared in three independent batches. For each batch, three electrodes were fabricated on different days following the same modification procedure. Each electrode was then incubated with 100 U/mL CA15-3 under identical conditions, and three consecutive DPV measurements were recorded. As shown in Figure 12d, the RSD values for batches 1–3 ranged from 0.625% to 1.850%, demonstrating good inter-batch consistency of the E-IS responses.

The reusability of the E-IS was further investigated using the same treated glassy carbon electrode. After incubation with 100 U/mL CA15-3 and the first measurement, the electrode was immersed in 0.1 M glycine–HCl buffer (GHB, pH 2.5) for 2 min for regeneration, then thoroughly rinsed with PBS to remove the bound analyte and subjected to DPV measurements under the same conditions. The electrochemical responses were recorded over five regeneration–measurement cycles. As shown in Figure 12e, the E-IS retained about 95% of its initial signal after five cycles, indicating good reusability.

These results show that the E-IS exhibits good precision, stability, selectivity, inter-batch consistency, and reproducibility of electrode fabrication, and thus holds promise for practical applications in CA15-3 detection.

### 3.7. Spiked Recovery Experiments and Actual Sample Analysis

For examining the applicability of the developed immunosensor, spiked recovery experiments were carried out. Normal human serum was diluted 1000-fold with PBS, and CA15-3 standard solutions were prepared at 0.01 U/mL, 10 U/mL, and 200 U/mL. The results of the measurement obtained using the constructed immunosensor are summarized in Table 1. As shown, the recovery rates of the spiked samples were between 100% and 119%, with corresponding RSDs between 0.13% and 0.49%. These results show that E-IS has good feasibility and accuracy in CA15-3 detection.

As illustrated in Table 2, ten samples of blood were collected from patients with BC to test the feasibility of the proposed E-IS for the measurement of CA15-3. All ten patients had serum CA15-3 levels above the normal human value of 25 U/mL, with values ranging from 27.47 U/mL to 262.12 U/mL, with RSDs between 0.35% and 5.42%. These results showed that the fabricated E-IS is capable of accurately measuring the serum CA15-3 biomarker related to BC.

### 3.8. Comparison of Different Sensors

A comparison between the immunosensor developed in this work and previously reported CA15-3 immunosensors is summarized in Table 3 In serum samples, the proposed device achieves a detection limit of 0.0031 U/mL and a linear range of 0–400 U/mL, with a correlation coefficient of R = 0.9979, a relative standard deviation (RSD) of 3.97%, and a signal retention of approximately 90% over 14 days (*n* = 3). These results indicate that the sensor offers a favorable combination of detection limit, working range, and precision, while maintaining a relatively simple operating procedure, which is meaningful for routine use in laboratories dealing with complex clinical matrices and highly variable analyte activities.

The present platform relies only on a conventional glassy carbon electrode and a standard electrochemical workstation, without the need for additional optical components, whereas many reported sensors involve more elaborate preparation steps and instrument configurations. On the other hand, its long-term stability is slightly inferior to that of some previously reported systems, and the current number of clinical samples remains limited. Overall, the sensor strikes a balance between sufficient sensitivity and simplified hardware requirements, with good operational practicality. Further improvement of its stability and the implementation of large-scale clinical validation will be important directions for future work.

## 4. Conclusions

In this study, a novel electrochemical immunosensor was successfully constructed for the determination of CA15-3 in serum by modifying a glassy carbon electrode with a CS@AuNPs/ZIF-8/rGO composite. By combining CS@AuNPs, ZIF-8, and rGO, the synergistic effects of the individual components are effectively utilized. The bottom ZIF-8/rGO layer acts as a nanostructured scaffold with a large specific surface area and porous channels, forming a continuous conductive network and accelerating electron transfer. By enhancing the interfacial loading capacity and optimizing the interface structure, ZIF-8 allows more CS@AuNPs to be immobilized and thereby indirectly improves the interaction between Au nanoparticles and substrate molecules. The upper CS@AuNPs layer serves as the main biofunctional film, where AuNPs provide binding sites for antibodies and CS, with its good biocompatibility, helping to stably immobilize protein molecules. This cooperative configuration enables the sensor to achieve high antibody loading, efficient charge transfer, and satisfactory analytical performance. The unique advantages of this composite material significantly improve the performance of the sensor and provide a solid basis for the highly sensitive detection of CA15-3.

Experimental outcomes disclosed that the constructed E-IS had very good linearity in CA15-3 serum activity of 0–400 U/mL with r^2^ = 0.9979, which indicated excellent sensitivity. Importantly, the sensor was able to reach a LOD of 0.0031 U/mL, which is well below the normal range of CA15-3 activities in clinical testing. The as-prepared immunosensor also exhibited excellent selectivity. Long-term usage tests and interference tests verified that the sensor can effectively detect CA15-3 in complex biological samples without interference from other substances, with strong anti-interference ability.

Furthermore, the immunosensor was applied to human blood samples, and high recovery rates were obtained, which further confirmed its reliability and accuracy in practical applications. With the merits of high sensitivity, stability, and selectivity, the CS@AuNPs/ZIF-8/rGO composite-modified GCE E-IS developed in this work has great potential for real-time monitoring and early BC screening. It offers a cutting-edge platform for early diagnosis, disease monitoring, and tailored therapeutic design in BC management.

While the findings of this study are promising, several critical steps remain before the method can be adopted in clinical testing. Currently, the evaluation is still based on a limited number of serum samples. Future work should involve systematic validation in larger and more diverse patient cohorts. In addition, the present work employs a laboratory three-electrode system and a conventional electrochemical workstation. In the future, miniaturization of the sensing platform could be explored to improve portability and enable rapid on-site monitoring. The proposed sensor may also be extended to a multi-analyte format for the simultaneous detection of CA15-3 and other breast cancer biomarkers, such as CEA and HER2, which is expected to further enhance its clinical applicability.

## Figures and Tables

**Table 1:** Spiked recovery experiment (*n* = 6).

No.	Spiked Activity (U/mL)	$\mathrm{Mean}\;\mathrm{Measured}\;\mathrm{Value}\;(\bar{x}\pm s$, U/mL)	Recovery Rate (%)	RSDs (%) (*n* = 6)
1	0.01	0.012 ± 0.002	119.090	0.17%
2	10	10.065 ± 1.351	100.651	0.13%
3	200	208.995 ± 3.366	104.498	0.49%

**Table 2:** Actual sample measurement results (*n* = 6).

No.	Average Activity $(\bar{x}\pm s$, U/mL)	RSDs (%) (*n* = 6)
1	262.12 ± 1.719	2.18%
2	41.650 ± 0.437	1.05%
3	202.26 ± 2.915	1.44%
4	34.350 ± 0.374	1.09%
5	51.460 ± 0.181	0.35%
6	59.430 ± 0.358	0.60%
7	27.470 ± 0.538	1.96%
8	109.450 ± 1.977	1.80%
9	70.330 ± 2.803	3.99%
10	138.890 ± 3.820	5.42%

**Table 3:** Comparison of sensors for CA15-3 Detection.

Modified Material	Detection Method	Linear Range (U/mL)	LODs (U/mL)	Stability (% Retention, Time)	RSD (%)	Samples (*n*)	Reference
PtCoNDs	DPV	0.1~200	0.0114	97%, 12 days	3.52	5	[31]
PMBCBImBr2-Zn-MOF	ECL	0.05~100	0.0275	94.3%, 14 days	5.10	3	[32]
MoS2	ECL	0.01~0.1	0.0039	93.3%, 18 days	NR	3	[33]
Au@Pt/Fe-CHO	ECL	0.5~200	0.17	98%, 12 days	4.50	5	[34]
CuS/rGO	DPV	1.0~150	0.3	95%, 28 days	2.70	5	[35]
Bi_2_O_3_/CdLa_2_S_4_/Bi_2_S_3_	EIS	0.001~100	0.0003	92.3%, 16 days	4.70	5	[36]
GO-PANI/ZAA	ECL	0.001~100	0.0003	97.4%, 200 s	NR	2	[37]
CS@AuNPs/ZIF-8/rGo	DPV	0.001~400	0.0031	90%, 14 days	3.97	10	This study

“NR” denotes not reported, Stability (% retention, time) represents the numerical value of stability maintenance and the testing time.

## Data Availability

The original contributions presented in this study are included in the article. Further inquiries can be directed to the corresponding author.

## Supplementary Materials

The following supporting information can be downloaded at: https://www.mdpi.com/article/10.3390/s25247462/s1, Figure S1: TEM images of the materials; Table S1: Serum dilution experiment.
