# Supplementary figures and images for: Electrochemical Immunosensor Based on CS@AuNPs/ZIF-8/rGO Composite for Detecting CA15-3 in Human Serum
Source: Sensors (Basel). 2025 Dec 8;25(24):7462. doi: 10.3390/s25247462 (PMC12736644; doi:10.3390/s25247462)

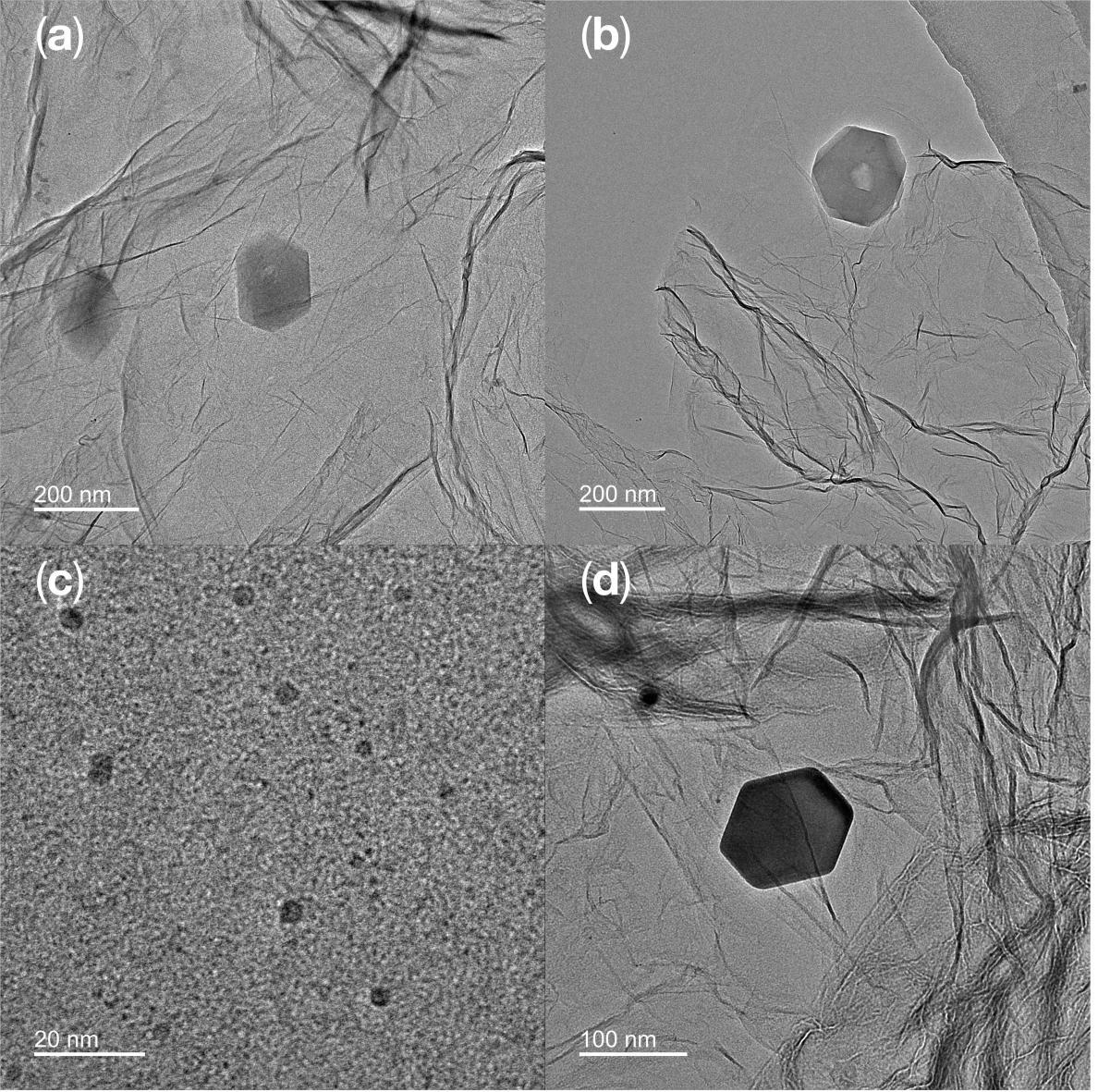

Supplement: Supplementary file 1 [file sensors-25-07462-s001.zip › Figure S1. TEM images of the materials.png]

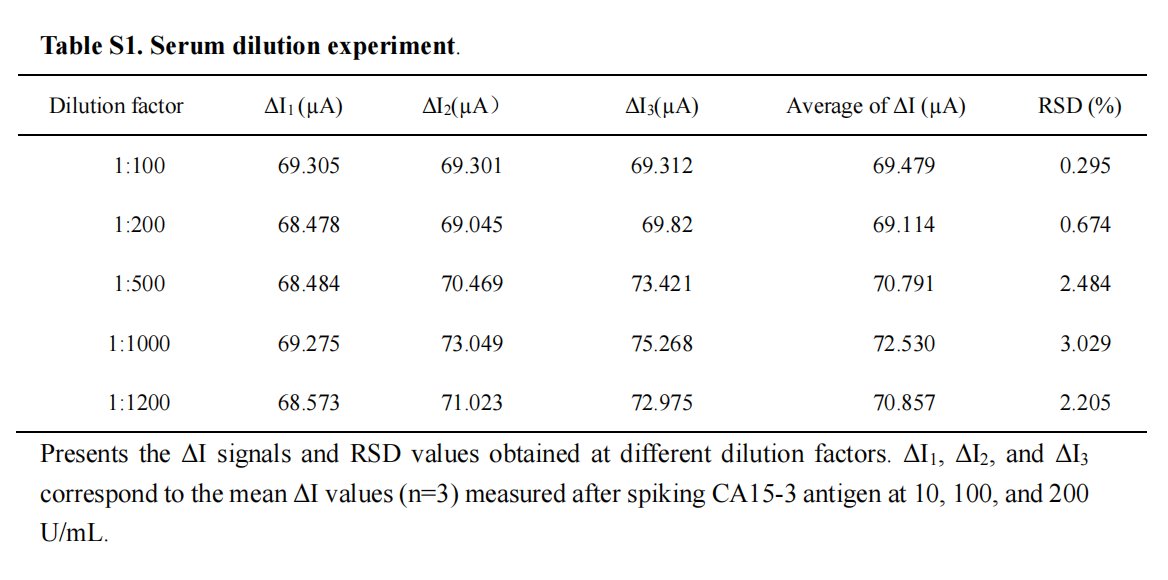

Supplement: Supplementary file 1 [file sensors-25-07462-s001.zip › Table S1.Serum dilution experiment.png]
